# Single-Molecule Microscopy Meets Molecular Dynamics Simulations for Characterizing the Molecular Action of Proteins on DNA and in Liquid Condensates

**DOI:** 10.3389/fmolb.2021.795367

**Published:** 2021-11-19

**Authors:** Kiyoto Kamagata

**Affiliations:** Institute of Multidisciplinary Research for Advanced Materials, Tohoku University, Sendai, Japan

**Keywords:** single-molecule, molecular dynamics, sliding, target search, diffusion, intrinsically disordered protein, liquid-liquid phase separation

## Abstract

DNA-binding proteins trigger various cellular functions and determine cellular fate. Before performing functions such as transcription, DNA repair, and DNA recombination, DNA-binding proteins need to search for and bind to their target sites in genomic DNA. Under evolutionary pressure, DNA-binding proteins have gained accurate and rapid target search and binding strategies that combine three-dimensional search in solution, one-dimensional sliding along DNA, hopping and jumping on DNA, and intersegmental transfer between two DNA molecules. These mechanisms can be achieved by the unique structural and dynamic properties of these proteins. Single-molecule fluorescence microscopy and molecular dynamics simulations have characterized the molecular actions of DNA-binding proteins in detail. Furthermore, these methodologies have begun to characterize liquid condensates induced by liquid-liquid phase separation, e.g., molecular principles of uptake and dynamics in droplets. This review discusses the molecular action of DNA-binding proteins on DNA and in liquid condensate based on the latest studies that mainly focused on the model protein p53.

## Introduction

**FIGURE 1:**
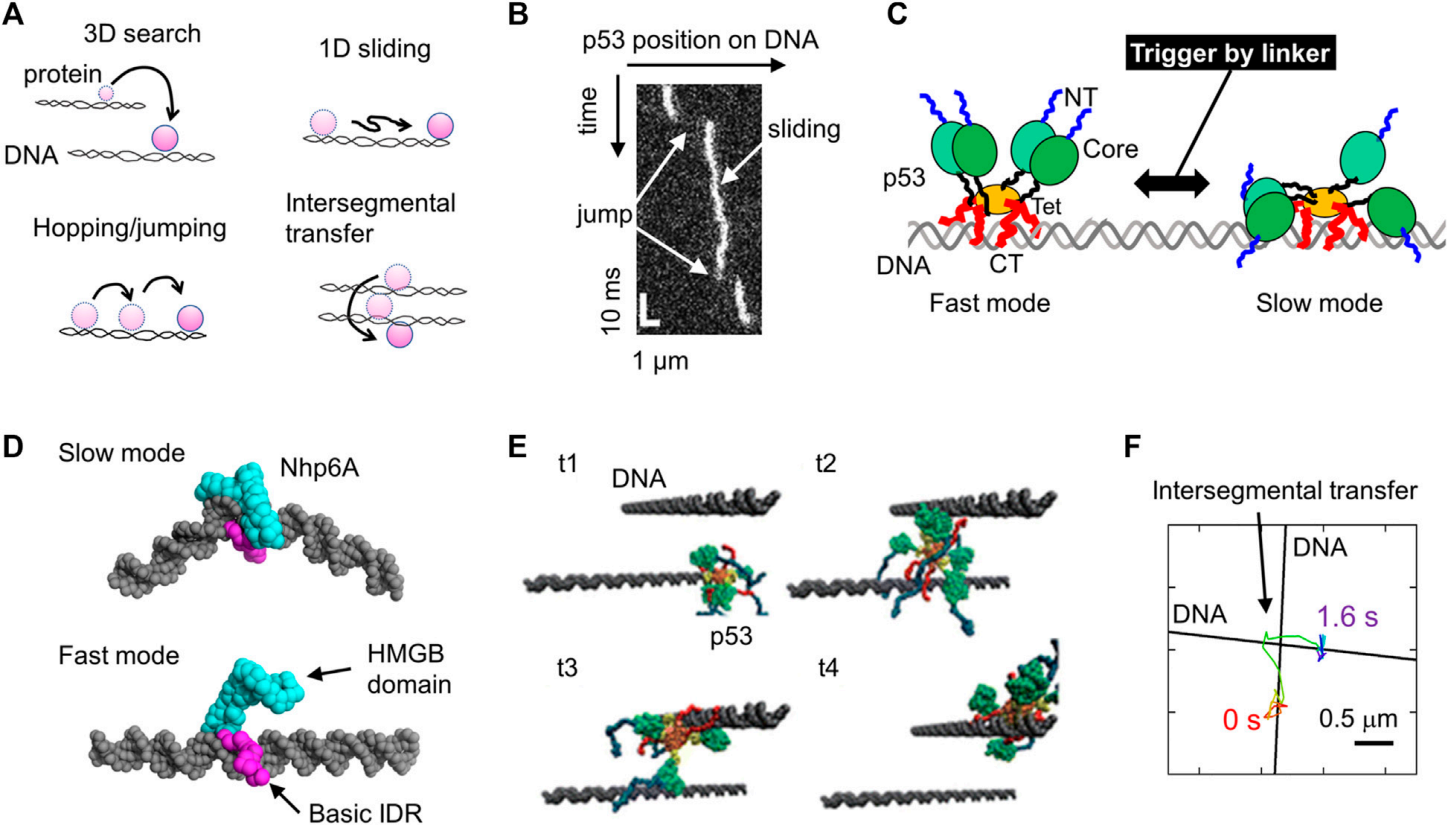
Molecular actions of DNA-binding proteins on DNA. **(A)** Four target search mechanisms of DNA-binding proteins, including 3D search in solution, 1D sliding along DNA, hopping and jumping along DNA, and intersegmental transfer. **(B)** Sliding and jumping of p53 along DNA as monitored using SM fluorescence microscopy. **(C)** Schematic diagram of the two sliding modes of p53 identified using SM fluorescence microscopy. p53 is composed of the NT (blue), Core (green), linker (black), Tet (orange), and CT (red) domains. NT and CT represent the N-terminal and C-terminal, respectively. The linker triggers the switch between the two modes. **(D)** Representative structures of two modes of Nhp6A as identified in coarse-grained MD simulations. **(E)** Intersegmental transfer trajectory of p53 in coarse-grained MD simulations. The time elapsed from t1 to t4. **(F)** Intersegmental transfer trajectory of p53 as observed via SM fluorescence microscopy. A p53 molecule transferred at the intersection between two DNA molecules. Panels (A-D, F) are adapted from various references (Itoh et al., 2018; Subekti et al., 2020; Kamagata et al., 2021b; Kamagata, 2021) with some modifications. Panel (E) was reprinted with permission from (Takada et al., 2015) Copyright 2015 American Chemical Society with some modifications.

DNA-binding proteins are key proteins that trigger or regulate cellular functions and determine cellular fate. They can trigger or regulate various reactions, such as transcription, DNA repair, and DNA recombination. Sequence-specific DNA-binding proteins function on target sites incorporated into genomic DNA. Before this, they need to search for and bind to their target sites in the genomic DNA. A failure of target binding causes a malfunction in DNA-binding proteins. Considering that 10^9^ base pairs (bps) of genomic DNA are much larger than 5–30 bps of target sites, DNA-binding proteins have gained a sophisticated target search mechanism that combines accuracy and speed under evolutionary pressure. Four search mechanisms have been proposed to date: 1) three-dimensional (3D) search with dissociation from and re-association with DNA, 2) one-dimensional (1D) sliding along DNA while maintaining contact with DNA, 3) hopping and jumping on DNA (short version of 3D search), and 4) intersegmental transfer between two DNA molecules (Figure 1A). The hopping and jumping are not well-distinguished because the criterion is not clear. An arbitrary distinction between them would be less than and more than e.g., 20 bp. Theoretical calculations demonstrate that the combined search, called facilitated diffusion, can reduce the search time significantly, up to a physiological time (second to minute time range) (Sheinman and Kafri, 2009; Bauer and Metzler, 2012; Veksler and Kolomeisky, 2013; Mahmutovic et al., 2015).

Single-molecule (SM) fluorescence microscopy can track molecules on DNA, which distinguishes between the four mechanisms and characterizes their details (Tafvizi et al., 2011b; Redding and Greene, 2013; Kamagata et al., 2017; Kamagata et al., 2020a; Park et al., 2021). Briefly, movements of fluorescently labeled proteins along stretched DNA have been recorded using an SM fluorescence microscope. Several methods have been developed to produce DNA arrays on the surfaces of coverslips (Yamamoto et al., 2000; Fazio et al., 2008; Igarashi et al., 2017). In addition, molecular dynamics (MD) simulations can reproduce the behavior of proteins on DNA in a computer, providing theoretical insight into the molecular actions that happen (Vuzman and Levy, 2012, 2014; Takada et al., 2015). Because of the high computational power required to simulate such large systems, coarse-grained models have been widely used; single residues are described as single beads and single nucleotides are as three or more beads, and the time evolution of molecules is calculated based on the interactions between two beads. These two approaches have intensively characterized and elucidated the molecular structure and dynamics of DNA-binding proteins. Furthermore, these methodologies have begun to characterize liquid-like droplets caused by liquid-liquid phase separation (LLPS), such as the molecular principles of uptake and dynamics in droplets (see details in *Liquid-Liquid Phase Separation*). This review discusses the molecular action of DNA-binding proteins on DNA and in liquid condensates based on the latest studies that mainly focused on the model protein p53.

## Action of DNA-Binding Proteins Along DNA

p53 is a multifunctional transcription factor that triggers cell cycle arrest, DNA repair, apoptosis, and suppresses uncontrolled cell proliferation (Beckerman and Prives, 2010; Bieging et al., 2014). p53 is also known to be the “guardian of the genome.” It forms a tetramer and utilizes a folded core domain and a disordered C-terminal (CT) domain for DNA binding in a specific and nonspecific manner, respectively (Anderson et al., 1997). Fifty percent of gene mutations in tumor cells were found in p53, and many identified mutations were located in the core domain, which prevents binding to the target DNA sequence (Joerger and Fersht, 2010). Since p53 has three common properties frequently observed in DNA-binding proteins, such as oligomerization, intrinsically disordered regions (IDRs), and multiple DNA-binding domains (Vuzman and Levy, 2012), this review mainly focuses on studies regarding p53.

### Sliding Along DNA

Tafvizi et al. provided direct evidence for the first time using SM fluorescence microscopy that p53 slides along DNA (Tafvizi et al., 2008). 1D sliding is described as bidirectional diffusion driven by thermal fluctuation (Figure 1B). Khazanov and Levy performed coarse-grained MD simulations of p53 and predicted that the basic disordered CT domains facilitate sliding dynamics (Khazanov and Levy, 2011). Almost simultaneously, this prediction was verified experimentally based on a comparison between wild-type p53 and a mutant lacking the CT domain (Tafvizi et al., 2011a). This study also proposed that p53 slides along DNA by hopping another DNA-binding domain, the core domain. This action of the core domain is reproduced in coarse-grained MD simulations (Terakawa et al., 2012). The sliding of p53 is slightly dependent on the DNA sequence (Leith et al., 2012), consistent with previous reports regarding DNA sequence-dependent interactions via core domains. Sliding is composed of two modes. p53 in fast mode can search for the target over a long sequence mainly using CT domains, whereas p53 can also recognize a DNA sequence using its core domain in slow mode (Murata et al., 2015; Murata et al., 2017) (Figure 1C). The transition between the two modes is facilitated by the interaction of the positively charged linker of p53 to DNA, recruiting core domains to DNA (Subekti et al., 2017) (Figure 1C). When p53 slides to its target site incorporated in a long DNA molecule, p53 recognizes and binds to the target with very low efficiency, and this recognition efficiency is regulated by a post-translational modification and disease-related mutation (Itoh et al., 2016). Together with the results of MD simulations (Terakawa and Takada, 2015), p53 passes over its target in most cases before a large conformational change upon target binding, resulting in low recognition efficiency.

Two molecular determinants of 1D sliding have been proposed based on studies on several DNA-binding proteins: basic IDRs as sliding promoters and intercalating residues as sliding suppressors. For the former, the basic IDR (CT domain) of p53 requires sliding, as described above (Tafvizi et al., 2011a; Murata et al., 2017). This is also supported by the fact that the designed peptide targeting the CT domain suppresses the sliding of p53 along DNA (Kamagata et al., 2019). Similarly, the deletion of basic IDRs from the nucleoid protein Nhp6A significantly slows its sliding along DNA, which was also assessed using coarse-grained MD simulations (Kamagata et al., 2021b). Nhp6A in fast mode slides along DNA, contacting the basic IDR without interaction of the folded HMGB domain with DNA, while Nhp6A tightly binds to DNA using the folded HMGB domain in slow mode (Figure 1C). Removing the basic IDR destabilizes Nhp6A in the fast mode, leading to motion through the slow mode. In addition, the basic IDR of Nhp6A enables the bypassing of obstacles bound to DNA (Kamagata et al., 2020c). The impact of basic IDRs on sliding dynamics is emphasized by engineering the genome-editing protein Cas9 by fusing it with the basic IDR from Nhp6A (Banerjee et al., 2021). For the latter, wedge residues in glycosylases (Phe residue in Fpg, Tyr residue in Nei, and Leu residue in *N*th) have been identified to intercalate into DNA bases, increasing the sliding friction of these proteins (Dunn et al., 2011; Nelson et al., 2014). Similarly, removing intercalating residues in Nhp6A (Met and Phe residues) facilitates its sliding along DNA (Kamagata et al., 2021b). Furthermore, the Arg residue in the nucleoid protein Fis significantly suppresses its sliding dynamics (Kamagata et al., 2021b). Thus, the combination of single-molecule microscopy and MD simulation has clarified some molecular principles of the sliding dynamics of DNA-binding proteins.

### Hopping and Jumping Along DNA

The hopping and jumping of p53 along DNA have been observed in coarse-grained simulations (Khazanov and Levy, 2011). However, the limited time resolution of SM microscopy (33 ms in typical cases) prevents verification of these processes. Recently, Subekti et al. improved the time resolution of SM microscopy to 0.5 ms and succeeded in detecting the jumping events of p53 along DNA (Subekti et al., 2020) (Figure 1B). The basic disordered CT domains of p53 are required for jumping (Subekti and Kamagata, 2021). In addition, a high-speed tracking of p53 demonstrated that p53 moves along DNA faster as the salt concentration increases. Salt dependence can distinguish sliding with continuous contact with DNA and hopping with short-term dissociation. This is because high salt concentrations do not affect sliding, but it facilitates hopping due to the weakened electrostatic interactions between p53 and DNA. Accordingly, the above results indicate the hopping dynamics of each DNA-binding domain of p53 on DNA at high salt concentrations. Hopping and jumping dynamics have been observed in other DNA-binding proteins such as the DNA polymerase UL42 (Komazin-Meredith et al., 2008), T7 DNA polymerase (Etson et al., 2010), the restriction enzyme EcoRV (Bonnet et al., 2008), and the nucleotide excision repair protein XPC-RAD23 (Cheon et al., 2019).

### Intersegmental Transfer Between Two DNA Molecules

Intersegmental transfer of p53 was also predicted using coarse-grained MD simulations (Khazanov and Levy, 2011; Takada et al., 2015) (Figure 1E). In these simulations, two DNA molecules were aligned close to each other, and the transfer of a p53 molecule was monitored. The p53 molecule first associated with a DNA molecule, captured a second DNA molecule while maintaining interaction with the first one, and then dissociated from the first one, resulting in a transfer between the two DNA molecules. Itoh et al. later provided experimental evidence of the intersegmental transfer of p53 using a fluorescence stopped-flow apparatus and SM microscopy (Itoh et al., 2018). SM microscopy enabled direct tracking of p53 molecules at the intersection of two DNA molecules, and mutational analysis revealed the involvement of the disordered CT domains (Figure 1F). Interestingly, the preceding simulations perfectly predicted the presence of intersegmental transfer and the involvement of the disordered CT domains. Intersegmental transfer has also been found in other DNA-binding proteins, including Fis and several transcription factors (Sox2, HoxD9, and Egr-1) (Iwahara and Clore, 2006; Iwahara et al., 2006; Takayama et al., 2010; Takayama and Clore, 2012; Esadze et al., 2014; Giuntoli et al., 2015). Among these DNA-binding proteins, the transfer rate of p53 was the highest. The architecture of p53, having four sets of disordered DNA-binding domains, may achieve efficient intersegmental transfer.

### Summary of the Molecular Activity of DNA-Binding Proteins

In this section, the molecular actions of DNA-binding proteins are discussed, mainly focusing on the model protein p53. These motions are categolized into passive ones driven by thermal fluctuation. Another type of the actions, observed in DNA translocation, is unidirectional motion of molecules along DNA, which is drived by ATP hydrolysis (Lee et al., 2014). SM microscopy and MD simulations have contributed to the molecular characterization of DNA-binding proteins. These methodologies can be applied to investigate more complicated targets, including the actions of protein complexes with multiple components and molecular communications between multiple proteins on DNA in the future.

## Liquid-Liquid Phase Separation

**FIGURE 2:**
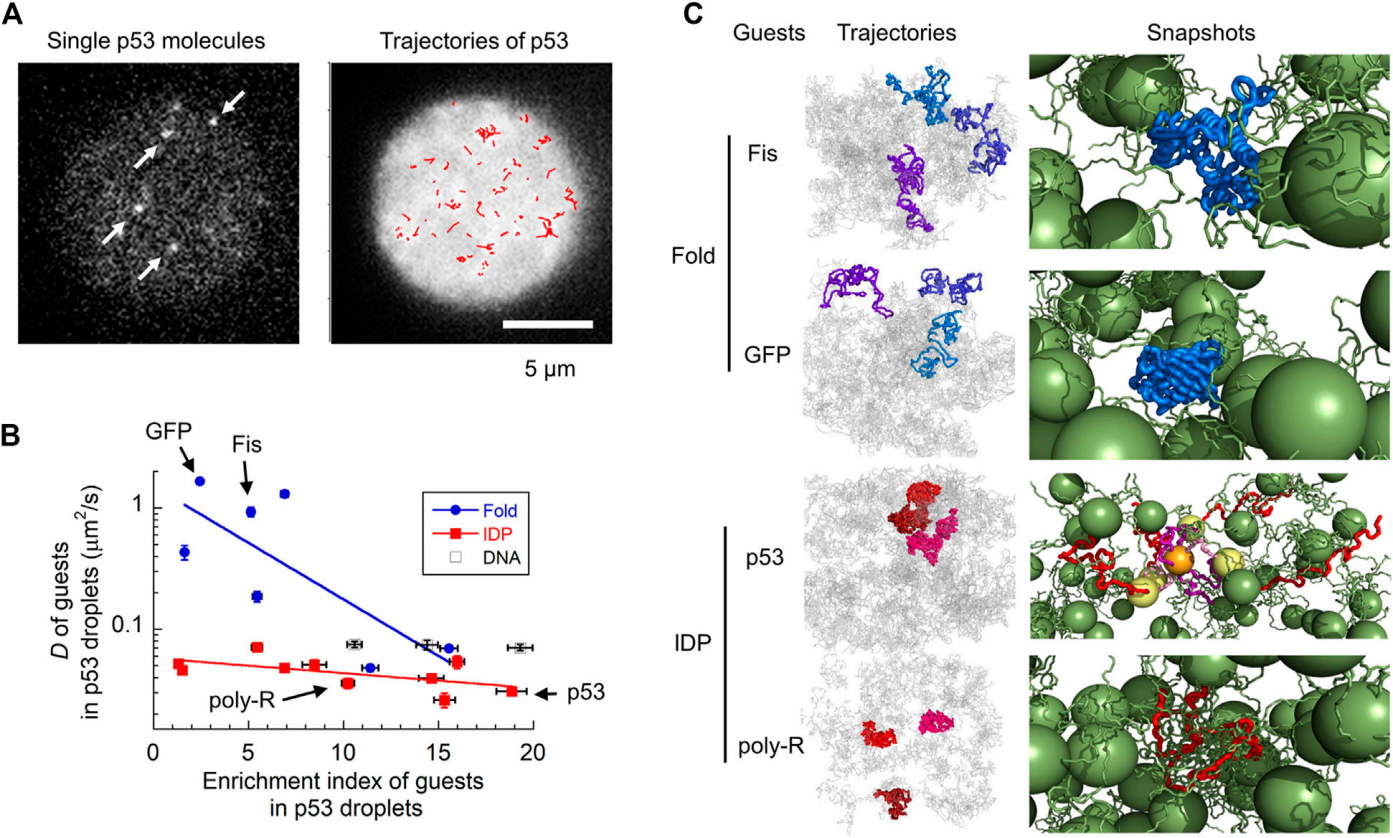
Molecular characterization of guest proteins in p53 droplets. **(A)** Snapshots and trajectories of guest p53 molecules in p53 droplets detected via SM fluorescence microscopy. The trajectories of molecules (red) in the right panel are plotted in a droplet (white). **(B)** Uptake preference (enrichment index) versus dynamics (diffusion coefficient) of various guest proteins and DNAs in p53 droplets revealed using SM fluorescence microscopy. The solid lines are for guiding folded proteins and IDPs using the eye. **(C)** Trajectories and snapshots of four guest proteins (Fis and GFP as folded proteins; p53 and poly-R as IDPs) in p53 droplets in coarse-grained MD simulations. In the snapshots, guest proteins are colored blue and red (as well as yellow and orange), whereas host p53 molecules are colored green. Panels **(A–C) **are adapted from (Kamagata et al., 2021c) with some modifications.

A more complicated example is in the functional condensates of biomolecules in non-membrane-bound organelles, including stress granules and nucleoli (Banani et al., 2017; Shin and Brangwynne, 2017; Darling et al., 2018; Larson and Narlikar, 2018; Feng et al., 2019). Proteins and RNAs assemble to form a liquid condensate (droplet) phase, separating themselves from a dilute bulk phase (Figure 2A). This phenomenon is also called LLPS. LLPS enables a wide variety of cellular functions and their regulation at levels that cannot be achieved by the dilute bulk phase alone. These functions include transcription, DNA condensation, and DNA repair. Many LLPS-related proteins have since been identified (Nott et al., 2015; Wei et al., 2017; Wang et al., 2018; Kamagata et al., 2020b). Earlier studies demonstrated that weak multivalent interactions between molecules are required to form liquid droplets. This review introduces what we have learned so far about the molecular grammar behind LLPS as deduced from MD and SM studies.

MD simulations have been widely used to characterize the behavior of molecules within liquid droplets (Dignon et al., 2018; Dignon et al., 2019; Garaizar et al., 2020; Hazra and Levy, 2020; Martin et al., 2020; Chu and Wang, 2021; Farr et al., 2021; Hazra and Levy, 2021; Lichtinger et al., 2021; Zhang et al., 2021). Because of the extremely high computational power required to simulate large LLPS systems, coarse-grained simulations have been developed and used. Coarse-grained models are simple compared to all-atom models, but they can reproduce distinctive features of liquid droplets: the concentrations of low- and high-density phases (Dignon et al., 2018), the critical temperature for phase separation (Martin et al., 2020; Lichtinger et al., 2021), temperature-dependent droplet formation (Dignon et al., 2019), and reentrant condensate phase in a high-salt environment (Krainer et al., 2021). The reentrant condensate phase means that the liquid condensate dissolves in a moderate salt concentration, but the condensate phase re-appears in a high salt concentration. These simulations have also predicted some properties of liquid droplets (e.g., relation between the temperature where a single chain collapse via intramolecular interactions and the critical temperature of phase separation (Dignon et al., 2018)), which might be worth testing experimentally in the future. In addition, all-atom simulations have characterized key residue-residue interactions in liquid droplet formation and regulation more precisely than coarse-grained simulations (Kamagata et al., 2021a; Krainer et al., 2021). For example, when coarse-grained simulations of liquid droplets were conducted, the relative interaction strengths between amino acid pairs in the coarse-grained model were modulated so as to fit all atom model (Krainer et al., 2021). These theoretical studies, therefore, highlight the validity of MD simulations for characterizing LLPS.

The combination of SM microscopy with MD simulation clarified the molecular grammar principles governing the LLPS (Kamagata et al., 2021c) (Figure 2). The localization and dynamic properties of guest proteins in liquid droplets have already been investigated using SM fluorescence microscopy. A series of guest proteins with different sizes, structures, and oligomeric states have been reported to be measured in host p53 droplets. Interestingly, molecular uptake did not significantly depend on the structural properties of the guest proteins. Although intrinsically disordered proteins (IDPs) tend to form liquid droplets as hosts (Brady et al., 2017; Vernon et al., 2018; Wang et al., 2018; Murthy et al., 2019; Kamagata et al., 2020b; Hardenberg et al., 2020; Krainer et al., 2021), guest IDPs do not exhibit any significant localization preferences. In contrast, the molecular uptake was moderately correlated with the total charge and number of R and Y residues. This demonstrates the impact of electrostatic and cation–π interactions in molecular uptake, consistent with the results obtained for host FUS droplets (Wang et al., 2018).

The dynamic properties of guest proteins have been investigated by tracking single molecules in droplets (Figure 2A) (Kamagata et al., 2021c). Contrary to their structure-indistinguishable recruitment property, the diffusion dynamics of guest IDPs are similar to that of the host p53, whereas those of folded proteins vary widely (Figure 2B). For example, folded proteins such as GFP and Fis diffuse much faster than IDPs, such as poly-R and p53. In addition, folded proteins show heterogeneous dynamics, consistent with biphasic dynamics (Niewidok et al., 2018) and hopping diffusion (Shayegan et al., 2019) observed in different samples. Single-molecule tracking of molecules within droplets can directly characterize the type of diffusion dynamics observed: superdiffusion, normal diffusion, and subdiffusion. Interestingly, all guest proteins exhibited normal diffusion in p53 droplets in the sub-second time range. Coarse-grained MD simulations reproduced the structure-dependent dynamics of guest proteins in p53 droplets (Figure 2C, left panels). These simulations demonstrated that folded proteins diffuse within the voids of the liquid droplet while interacting weakly with neighboring host proteins, leading to fast diffusion (right panels of Fis and GFP, Figure 2C). The host proteins became non-uniformly distributed, creating voids at the microscopic level (Wei et al., 2017; Schuster et al., 2018; Cinar et al., 2019), causing complicated dynamics of the guest proteins. In contrast, IDPs adapt their structures to form tight interactions with the host proteins, resulting in slow diffusion (right panels of p53 and poly-R, Figure 2C).

Thus, this combined approach sheds light on the molecular grammar of LLPS. It would be necessary to examine whether the structure-dependent dynamics in droplets are true in different protein systems. The molecular characterization of LLPS is still in its infancy and is expected to be further examined in the future in combination with other methodologies, including SM fluorescence resonance energy transfer and NMR.

## Summary and Future Perspectives

The combination of SM microscopy with MD simulations has provided a molecular understanding of DNA-protein interactions and LLPS. The results obtained by SM experimentalists have motivated MD theoreticians to challenge subsequent studies and vice versa. This review focused on studies of the model protein p53, but the two approaches could also be applied to other DNA-binding proteins or LLPS-related molecules to characterize the molecular actions of their individual targets. In addition, the molecular characteristics and principles revealed by these studies could lead to the engineering of biomolecules and the design of artificial regulators in the future.
